# Nanoscale true random bit generator based on magnetic state transitions in magnetic tunnel junctions

**DOI:** 10.1038/s41598-019-52236-z

**Published:** 2019-10-30

**Authors:** Alex S. Jenkins, Lara San Emeterio Alvarez, Paulo P. Freitas, Ricardo Ferreira

**Affiliations:** International Iberian Nanotechnology Laboratory, INL, Av. Mestre José Veiga s/n, 4715-330 Braga, Portugal

**Keywords:** Spintronics, Magnetic devices

## Abstract

We present an investigation into the in-plane field induced free layer state transitions found in magnetic tunnel junctions. By applying an ac current into an integrated field antenna, the magnetisation of the free layer can be switched between the magnetic vortex state and the quasi-uniform anti-parallel state. When in the magnetic vortex state, the vortex core gyrates a discrete number of times, and at certain frequencies there is a 50% chance of the core gyrating n or n − 1 times, leading to the proposal of a novel nanoscale continuous digital true random bit generator.

## Introduction

As modern society evolves, and more and more data is being constantly shared through technology’s increasing interconnectivity, the need for cryptographic solutions continues to be an increasingly pressing issue. An integral part of the hardware of modern cryptography is a true random number generator, which is scalable, energy efficient and reliable. Nanotechnology solutions for true random number generators have been proposed previously, including using random telegraph noise^1^, semiconductor lasers^2^ and diffusive memristors^3^. Whilst the different systems all have relative advantages and drawbacks, there remains common issues with scalability, reliability and systematic bias due to environmental factors, such as temperature, which require complicated post-processing. In this paper, a high frequency robust true random number generator with a resistive output, nanometric footprint and potential to be engineered towards ultra-low power consumption and direct CMOS integration is presented, with the potential to make meaningful impact for IoT cyber-security applications.

**Figure 1:**
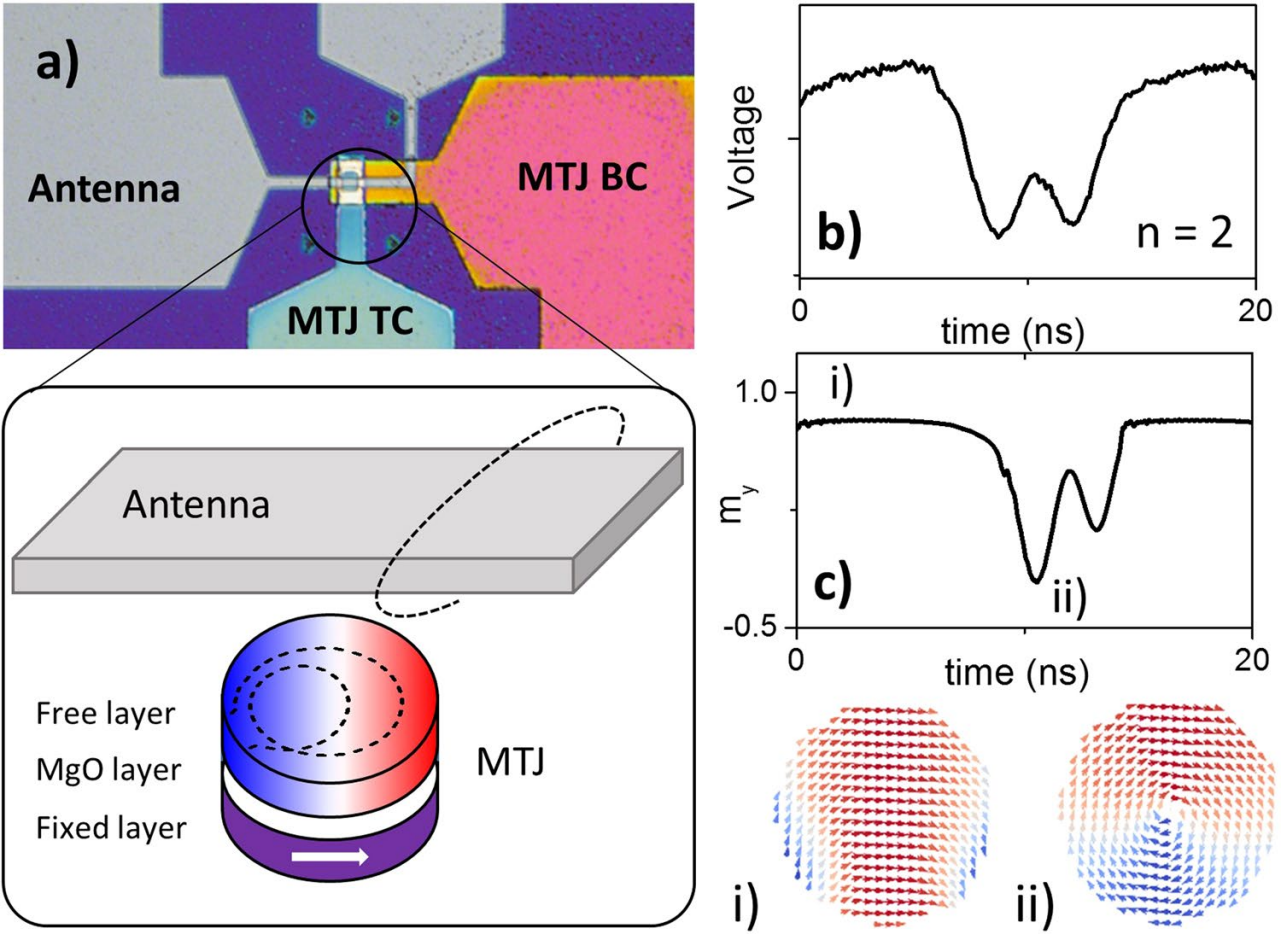
(**a**) micrograph of device with the MTJ contacted via a top and bottom contact (TC and BC) with an antenna integrated above. The schematic shows MTJ and antenna, the dashed line on the MTJ represents the trajectory of the vortex core calculated from micromagnetic simulations. (**b**) voltage measured at ${{\boldsymbol{I}}}_{{\boldsymbol{MTJ}}}^{{\boldsymbol{dc}}}$ = −8 mA and (**c**) magnetisation component along the y-axis for an excitation frequency of 70 MHz, with examples of the magnetisation configuration of (i) the QUAP state and (ii) the vortex state.

One exciting avenue of research for emerging nanotechnology solutions is the field of spintronics. One of the main building blocks of spintronics is the magnetic tunnel junction (MTJ), where a free magnetic layer is separated from a fixed magnetic layer by an insulating layer, as shown schematically in Fig. 1a. Some of the major reasons behind MTJs attracting significant interest is due to their relative durability, large magnetoresistance values, nanoscale and compatibility with CMOS integration. MTJs are already used for a variety of applications, such as conventional^4^ and bio-inspired^5–9^ memories, magnetic hard drive read heads^10,11^, magnetic field sensors^12^ and high frequency wireless components^13–23^, and have recently been proposed as a so called ‘spin dice’, where the stochastic nature of the switching has been utilised as a true random number generator^24^.

## Methods

In this report we present data on a 300 nm diameter magnetic tunnel junction with a stack 5 Ta/50 CuN/5 Ta/50 CuN/5 Ta/5 Ru/6 IrMn/2.0 CoFe30/0.7 Ru/2.6 CoFe40B20 /MgO/2.0 CoFe40B20/0.2 Ta/7 NiFe/10 Ta/7 Ru (thicknesses in nanometers), annealed under at 330 C for 2 h and cooled down under an in-plane magnetic field of 1 Tesla. The free layer is in the vortex ground state^13^ and can be modulated with an in-plane magnetic field, between the vortex state and a magnetisation state which is quasi-uniform, i.e. the magnetisation is predominantly along a specific direction, but with some slight tilting at the edges characteristics of a C-state. The magnetic vortex state corresponds to a closed loop of in-plane magnetisation with a central out-of-plane vortex core. The MTJ nano-pillars produced have a DC resistance of 55 Ohm in the anti-parallel state and a DC resistance of 48 Ohm when a vortex core is centred in the pillar.

The in-plane magnetic field is produced by an integrated 300 nm thick Al antenna with a width of 3 µm positioned approximately 600 nm above the nanopillar, through which dc and electrical current is passed. The dc current is applied so as to shift the vortex core closer to the edge of the pillar and determines the direction of the magnetisation after the vortex has been expelled from the nanopillar. If a positive current is applied, this favours the quasi-uniform parallel state (QUP) and if a negative current is applied the quasi-uniform antiparallel state (QUAP) is favoured.

In addition to the dc current, a simultaneous ac current is also passed across the antenna, which generates a time varying in-plane magnetic field collinear to the reference layer of the MTJ. The combination of the ac and dc components of the in-plane magnetic field results in the free layer constantly switching between the vortex state and the QUAP state, as shown in Fig. 1b,c. The net current, which is a combination of the ac and dc current, is such that when the ac current is at a maximum, the free layer enters the QUAP state, and when the ac current is at a minimum the free layer enters the vortex state.

In Fig. 1b, the voltage is measured at a constant dc current across the MTJ, and shows how the resistance of the MTJ varies as a function of time due to the tunnelling magnetoresistance effect. The micromagnetic simulations, shown in Fig. 1c, well confirm this behaviour and demonstrate how this behaviour can be understood as the free layer transitioning between the two magnetic states (i.e. the vortex and QUAP states). For this measurement, a dc current of ${I}_{MTJ}^{dc}$ = −8 mA is applied to the MTJ in order to measure the voltage and ${I}_{Ant}^{dc}$ = −40 mA is applied to the antenna. The ac current applied to the antenna is ${I}_{Ant}^{ac}$ = 46 mA (${P}_{Ant}^{ac}$ = 31 mW), with a frequency of ${f}_{Ant}^{ac}$ = 70 MHz. Throughout the measurement a perpendicular field of H_perp_ = 0.4 T is applied to the device. This field is applied in order to reduce the hysteresis in the switching between the vortex state and the QUAP state. Other options for modifying the hysteresis of the device include changing the thickness and diameter of the free layer, as well as designing non-symmetric nano-pillars (i.e. introducing a notch nucleation site).

The micromagnetic simulations were performed using the mumax3 code^25^ and the following parameters: Ms = 680e3 A/m, Aex = 1.2e11, alpha = 0.01, Bz = 0.4 T, thickness = 9 nm, diameter = 400 nm, j_dc_ = −40e9, Temp = 0 K, ${B}_{y}^{dc}$ = 20 mT and ${B}_{y}^{ac}$ = 15 mT.

## Results

In Fig. 1b,c, the free layer is initially in the QUAP state, corresponding to a plateau in the voltage (or correspondingly the resistance) as the magnetisation component collinear to the fixed layer, m_y_, is saturated. When the ac field is at a minimum the free layer transitions into the vortex state, and the vortex core moves in a circular orbit at a resonant frequency around 260 MHz, which corresponds to the fundamental mode for vortex oscillators known as the gyrotropic mode^13^. With an excitation frequency of 70 MHz applied there is time for a total of two gyrations of the vortex core, i.e. n = 2, before the core is re-expelled. In Fig. 1a, the trajectory of the vortex core, calculated from the micromagnetic simulations, is plotted on the schematic of the MTJ, and can be seem to be roughly circular.

**Figure 2:**
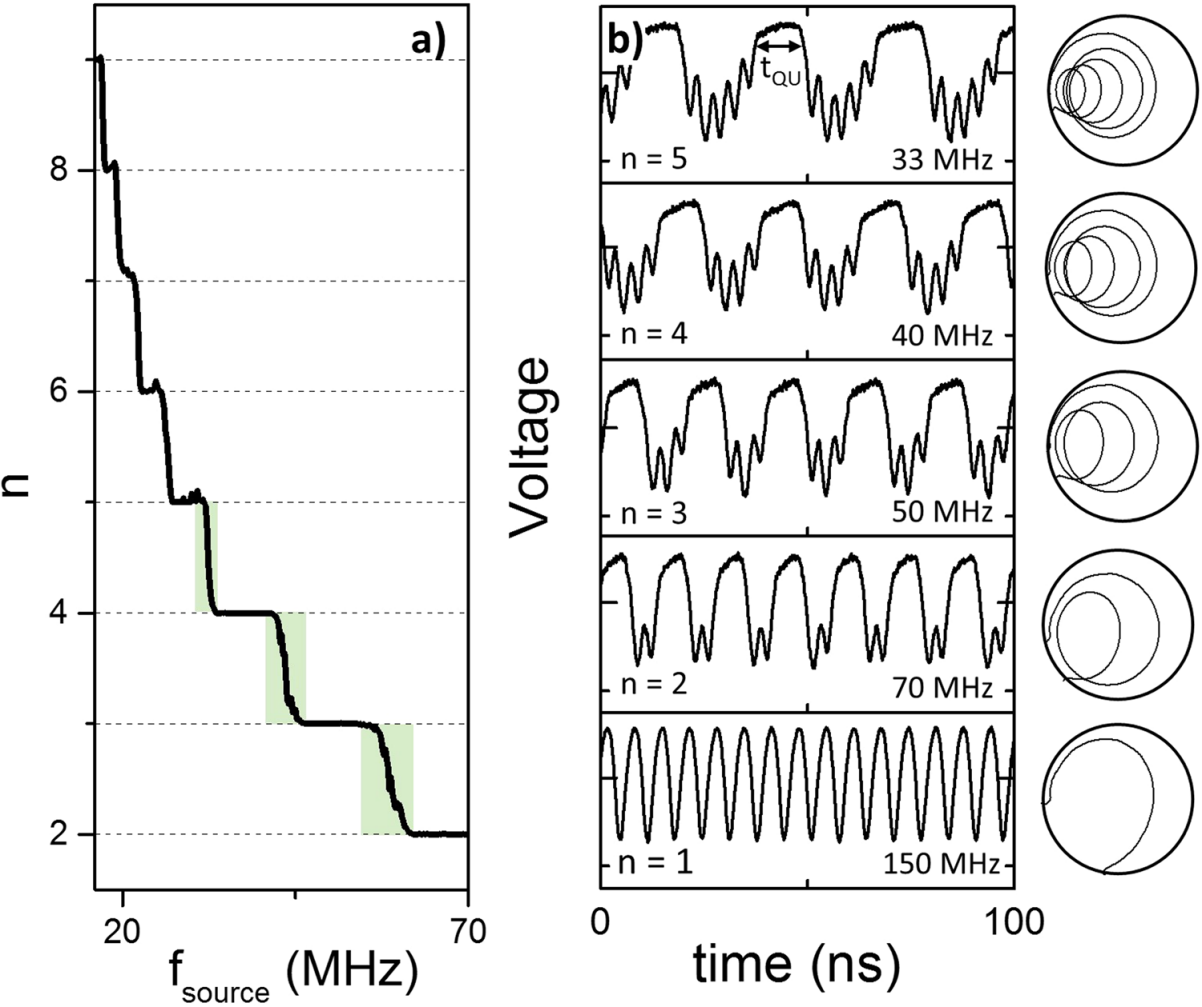
(**a**) number of gyrations, n, versus frequency and (**b**) the measured voltage as a function of time for five different excitation frequencies showing the discrete number of vortex core gyrations before expulsion, n. The trajectories of the vortex core on the right hand side are calculated from micromagnetic simulations.

In Fig. 2 we expand upon the frequency dependence of the transitions between the QUAP state and the vortex state. By analysing the measured voltage in the time domain, the number of gyrations which the vortex core performs over a 5 µs window can be extracted and is plotted as a function of the excitation frequency in Fig. 2a. There is a clear discretisation of the number of gyrations which the vortex core performs before being expelled by the ac field.

In order to expand upon this discrete dynamic behaviour, in Fig. 2b the measured voltage in the time domain is plotted for a selection of excitation frequencies and the number of gyrations observed, n, can clearly be seen to change as a function of the excitation frequency. The trajectories calculated via micromagnetic simulations for the corresponding excitation frequencies are plotted to the right of the figure and show the position of the vortex core at any given moment.

For lower frequencies, i.e. f_source_ = 33 MHz, the time for a single period of the field excitation is longer and as such the vortex core will gyrate a greater number of times before eventually being re-expelled. As the frequency is increased, there is less time spent in the vortex state, and as such there are fewer gyrations observed, until for sufficiently high frequencies, where only one single gyration is observed before the core is re-expelled. The time spent in the quasi-uniform state, t_QU_, depends upon the number of gyrations the vortex undergoes, and is different for each excitation frequency.

**Figure 3:**
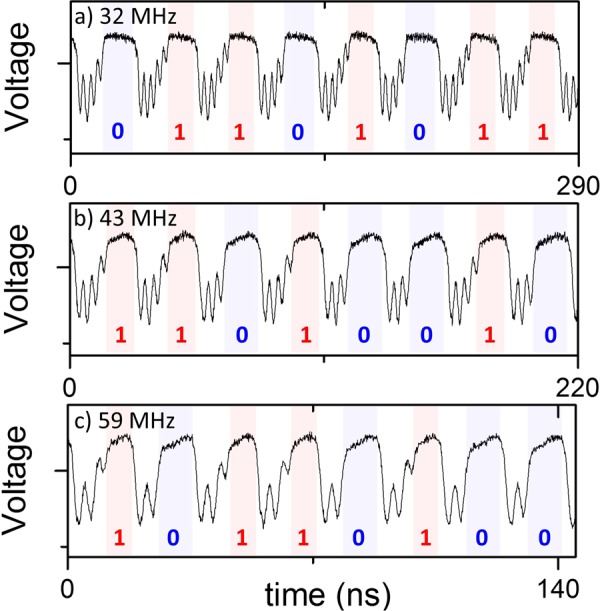
The measured voltage as a function of time at a given frequency of (**a**) 32 MHz, (**b**) 43 MHz and (**c**) 59 MHz. The shaded regions show time when free layer is in the QUAP state.

Having discussed the discrete nature of n in Fig. 2, in Fig. 3 we discuss what happens at frequencies which are between two discrete values of n, highlighted in the green shaded area in Fig. 2a. At these frequencies there is a finite probability of the vortex either gyrating n or n − 1 times before it is re-expelled. This can be seen in Fig. 3a–c where the vortex gyrates either 5 or 4 times, 4 or 3 times and 3 or 2 times, respectively. This stochastic behaviour is related to the trajectory of the vortex core. In Fig. 2, the trajectories for different values of n are plotted, and it can be seen that the vortex core passes close to the edge of the nanopillar, which is where the annihilation of the vortex core takes place during the vortex expulsion. For certain frequencies, the vortex core passes sufficiently close to the edge of the pillar such that there is a probability of the core annihilating and a probability that it will continue gyrating for another turn.

The amount of time that the vortex spends in the QUAP state depends upon the time at which the vortex core is expelled. The regions where the free layer is in the QUAP state have been shaded in Fig. 3. In Fig. 3a, the free layer will gyrate either 5 or 4 times, and will correspondingly spend either 18.0 µs or 14.2 µs, respectively, in the QUAP state. An analogous situation arises for Fig. 3b,c as well, with the free layer spending either 13.6 µs or 10.6 µs (Fig. 3b) and 9.2 µs or 7.8 µs (Fig. 3c) depending upon the number of gyrations which the free layer makes when in the vortex state. By simply measuring the time spent in the QUAP state, for example with a basic voltage integrator circuit, a digital random bit will be constantly generated over a finite period of the order of tens of nanoseconds.

**Figure 4:**
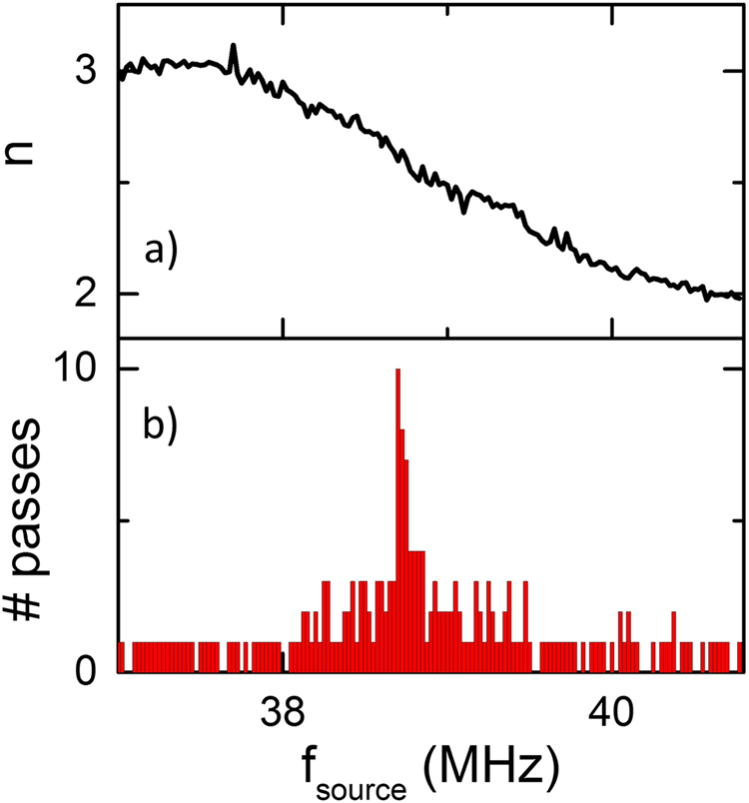
(**a**) number gyrations and (**b**) # number of NIST tests passed as a function of the excitation frequency.

It is crucial for a true random number generator to be truly random, a characteristic which can be statistically tested using the NIST tests^26^. In Fig. 4, the voltage was measured in the time domain using a dc current of ${I}_{MTJ}^{dc}$ = −3 mA to the MTJ and ${I}_{Ant}^{dc}$ = −40 mA to the antenna, with an ac current ${I}_{Ant}^{ac}$ = 36 mA (${P}_{Ant}^{ac}$ = 19 mW) also applied to the antenna. The voltage measured across the MTJ over a 500 µs window has been converted into a string of digital bits by partitioning the measurement into small windows equal to the inverse of the excitation frequency, i.e. t_window_ = 1/f_source_, and integrating, so that n = 3 is converted in to a 1 and n = 2 is converted into a 0. This digital string is then subjected to 10 NIST tests: monobit, frequency within block, runs, longest run ones in block, dft, non-overlapping template matching, overlapping template matching, serial, approximate, cumulative sums, random excursion and random excursion variant test. It should be noted that the number of tests which could be implemented was limited by the length of the bit string around 20,000 bits. In Fig. 4a the number of gyrations is plotted as a function of the frequency as well as (b) the corresponding number of test passes. It can be seen that when there is roughly a 50% chance of the vortex core gyrating 2 or 3 times, i.e. f_source_ = 38.7 MHz, all 10 random tests are passed.

The data shown in Fig. 4, shows that the vortex based TRNG has the potential to generate a continuous stream of random bits at a rate of 38.7 Mbits/s (compared to 0.6 Mbits/s for the spin dice^24^), without the need for a reset step. In fact many MTJs could be excited by a single field line, which means that N number of synchronised TRNGs could be operating continuously in parallel. Furthermore the probability of the bits can be easily modulated by applying a modulated excitation signal, due to the dependence of the probability with frequency.

In conclusion, we present a novel nanoscale digital true random number generator based on vortex to quasi-uniform transitions in magnetic tunnel junctions. The number of gyrations is discretised, and at a certain excitation frequency there is an equal probability of n or n − 1 gyrations. By integrating the resultant voltage signal a continuous digital nanoscale nanosecond true random number generator can be envisaged, which is of potential interest for emerging cryptography applications.

## Data Availability

The data that support the findings of this study are available from the corresponding author upon request.
